# Associations between work-related variables and workplace violence among Chinese medical staff: A comparison between physical and verbal violence

**DOI:** 10.3389/fpubh.2022.1043023

**Published:** 2023-01-10

**Authors:** Long Sun, Wen Zhang, Aihua Cao

**Affiliations:** ^1^Centre for Health Management and Policy Research, School of Public Health, Cheeloo College of Medicine, Shandong University, Jinan, China; ^2^NHC Key Lab of Health Economics and Policy Research, Shandong University, Jinan, Shandong, China; ^3^Department of Psychiatry, Binzhou People Hospital, Binzhou, Shandong, China; ^4^Department of Pediatrics, Qilu Hospital of Shandong University, Jinan, Shandong, China

**Keywords:** physical violence, verbal violence, work-related variables, medical staff, China

## Abstract

**Background:**

Workplace violence (WPV) against medical staff has been an important public health and societal problem worldwide. Although numerous studies have implied the differences between physical violence (PV) and verbal violence (VV) against medical staff, few studies were conducted to analyze the different associations between work-related variables, PV, and VV, especially in China.

**Methods:**

A cross-sectional study was conducted among Chinese medical staff in public hospitals, and 3,426 medical staff were interviewed and analyzed. WPV, including PV and VV, were evaluated by the self-report of the medical staff. Work-related variables, physical disease, depression, and social-demographic variables were also measured. The work-related variables included types of medical staff, professional titles, hospital levels, managers, working years, job changing, working hours/week, night duty times/week, monthly income, self-reported working environment, and social position. Logistic regressions were conducted to examine the factors associated with PV and VV.

**Results:**

A total of 489 medical staff (23.0%) reported the experience of PV and 1,744 (50.9%) reported the experience of VV. Several work-related variables were associated with PV and VV, including nurse (OR = 0.56 for PV, *p* < 0.01; OR = 0.76 for VV, *p* < 0.05), manager (OR = 1.86 for PV, *p* < 0.01; OR = 1.56 for VV, *p* < 0.001), night duty frequency/week (OR = 1.06 for PV, *p* < 0.01; OR = 1.03 for VV, *p* < 0.01), bad working environment (OR = 2.73 for PV, *p* < 0.001; OR = 3.52 for VV, *p* < 0.001), averaged working environment (OR = 1.51 for PV, *p* < 0.05; OR = 1.55 for VV, *p* < 0.001), and bad social position (OR = 4.21 for PV, *p* < 0.001; OR = 3.32 for VV, *p* < 0.001). Working years (OR = 1.02, *p* < 0.05), job changing (OR = 1.33, *p* < 0.05), and L2 income level (OR = 1.33, *p* < 0.01) were positively associated with VV, but the associations were not supported for PV (all *p*>0.05). The other associated factors were male gender (OR = 1.97 for PV, *p* < 0.001; OR = 1.28 for VV, *p* < 0.05) and depression (OR = 1.05 for PV, *p* < 0.001; OR = 1.04 for VV, *p* < 0.001).

**Conclusion:**

Both PV and VV were positively associated with work-related variables, such as doctor, manager, more night duty frequency, perceived bad working environment, or social position. Some variables were only associated with VV, such as working years, job changing, and monthly income. Some special strategies for the work-related variables should be applied for controlling PV and VV.

## 1. Background

The World Health Organization (WHO) defined workplace violence (WPV) as “incidents where staff is abused, threatened or assaulted in circumstances related to their work” (1). In recent decades, several studies reported that more than 60% of medical staff have experienced WPV in the world (2–4). In China, the prevalence of WPV against medical staff appears to be on the rise in recent years (5, 6). In addition, previous studies also identified several negative outcomes of WPV, such as depressive symptoms, cardiovascular disease, and so on (7–9). We have enough reasons to conclude that WPV against medical staff has been an important public health and societal problem worldwide (10, 11), which should gain our attention.

Due to the importance of WPV, several studies had been conducted to explore the factors associated with WPV against medical staff, and several associated factors were identified, such as social-demographic (12, 13), psychological and physical health (9, 14–18), and work-related variables (19–22). For work-related variables, most of these studies focused on the association between work stress, work burnout, social support, work environment, and WPV (19–22). The work for medical staff was characterized by long working hours, frequent night duty, and a high workload (23, 24). However, only a few studies were conducted to explore the associations between these work-related variables and WPV (25–27). When we further reviewed these studies, most of them were conducted among special kinds of medical staff in China, such as general practitioners (28) and nurses (29). China is the country with the highest population and the most health services in the world (30), and the seriousness and social impact of WPV were also at a high level (6, 31). Thus, studies about the associations between work-related variables and WPV in China not only can help us to build associations with a wider range of medical staff but also can help us to supply the evidence to control WPV in the world.

In contrast, physical violence (PV) and verbal violence (VV) were the main classifications for WPV against medical staff. In recent years, studies have identified a higher prevalence of VV than PV among medical staff (32, 33). Another study also supported that nurses were at higher risk of PV and doctors were at higher risk of VV (34). In Turkey, one study supported that average working time per week was associated with PV but not with VV among nurses (35). All these findings implied that there may be some differences between PV and VV. However, fewer studies were conducted to explore the differences between PV and VV, especially in China.

To fill the gaps, we conducted a cross-sectional study among Chinese medical staff. In this study, our first aim was to explore the associations between work-related variables and WPV among a wider range of medical staff. The second aim was to explore the differences in work-related variables between PV and VV among medical staff. The findings help us better understand the associations between work-related variables and WPV in the world, and they can also provide us with special strategies to control PV and VV against medical staff.

## 2. Methods

### 2.1. Setting and participants

This is a cross-sectional study conducted among medical staff in Shandong Province, China. Shandong Province is located in the east of China, and its population ranks second among all the Chinese provinces (36). The number of health workers in Shandong province ranked first in all the Chinese provinces (37). In this study, we used multiple stratified random cluster sampling methods to recruit medical staff in general hospitals. First, all 17 cities in Shandong were divided into three levels based on the gross domestic product (GDP) per capita in 2018 (36), and one city was randomly selected from each level. Second, we randomly selected one municipal hospital from each of the selected cities. In this step, three counties (districts) were also randomly selected from each of the selected cities. Third, the general county-level hospitals (district-level hospitals) in the selected counties (districts) were chosen to conduct this study. Finally, we selected three municipal hospitals and nine county-level hospitals. In these hospitals, three inpatient areas from each department were randomly selected in municipal hospitals, and two inpatient areas from each department were randomly selected in county-level hospitals. Medical staff, including doctors, nurses, and medical technicians, who worked on the interview date were recruited to participate in the survey. We interviewed and analyzed 3,426 medical staff in this study. The ratios for sample size and analyzed variables should be higher than 5. In this study, we analyzed 17 variables, and the smallest analyzed sample size was 2,127, which is adequate for the data analyses.

### 2.2. Data collection

This study was conducted from December 2018 to January 2019. The questionnaires were sent to medical staff individually, and they filled them out anonymously. On the interview date, two trained postgraduate students were stationed in the hospital to answer the questions and collect the questionnaires. Totally, we trained eight postgraduate students to complete the survey in one city.

### 2.3. Measures

#### 2.3.1. Workplace violence, verbal violence, and physical violence

Workplace violence (WPV) was assessed by the question, “Have you ever experienced the following behavior conducted by your patients or their relations?” The answers were verbal violence, physical violence, both verbal and physical violence (BV), and no violence (NV). In this study, physical violence (PV) was recoded as yes (1) or no (0), with the former one including medical staff reporting physical violence and BV. VV was also recoded as yes (1) or no (0), with the former one including medical staff reporting verbal violence and BV. This question has been used to evaluate WPV in many previous studies (32, 38, 39).

#### 2.3.2. Work-related variables

In this study, the work-related variables contained types of medical staff, professional titles, hospital levels, managers, working years, job changing, working hours/week, night duty times/week, monthly income, self-reported working environment, and self-reported social position. Types of medical staff included doctors (1), nurses (2), and medical technicians (3). The professional title was evaluated by senior (1), vice-senior (2), intermediate (3), and junior and others (4). As this study was conducted among municipal and county-level hospitals, there was no level 1 hospital. Hospital level was measured by level 2 (0) and level 3 (1). The manager was measured by yes (1) or no (0), and the former one contained the dean/vice-dean, director/vice-director, and change nurse. Job changing was also evaluated by yes (1) or no (0), and medical staff who had full-time labor in other institutions were marked true for job changing. Working hours per week were evaluated by the question, “How many hours do you work per week on average?” The participants answered the number of hours per week that they worked. The number of working hours/week was analyzed in this study. Night duty frequency/week was measured by the average times of night duty for the participants. Income level was evaluated by the question about the income of the participants, including salary, bonus, and all the other kinds of income. The answers can be chosen from ≤ 3,000 RMB, 3,001–5,000 RMB, 5,001–7,000 RMB, 7,001–9,000 RMB, 9,001–11,000 RMB, 11,001–13,000 RMB, and ≥13,001 RMB. As fewer participants chose the last 3 answers, we recoded them as follows: ≤ 5,000 RMB (L1), 5,001–9,000 RMB (L2), and ≥9,001 RMB (L3). A US dollar is approximately equal to 7 RMB. The working environment was evaluated by the self-reported question, “What do you think about your current working environment?” The answer contained very good, good, average, bad, and very bad. We recoded them into good (1), average (2), and bad (3). The good classification contained very good and good options, and the bad classification contained bad and very bad options. Social position was also evaluated by the self-reported question, “What do you think about your current social position?” The answer also contained very good, good, average, bad, and very bad. We recoded them into good (1), average (2), and bad (3). The good classification contained very good and good options, and the bad classification contained bad and very bad options.

#### 2.3.3. Social-demographic variables

Gender was coded as male (0) and female (1). Age was calculated by the date of birth of the participants. Marital status was evaluated by single, married, divorced, widowed, and others. As the percentage of the last three answers was small, we recoded it as single (1), married (2), and others (3). Education was assessed by the academic degree received by the participants. The answers were doctor, master, bachelor, junior college, secondary specialized school, high school, middle school, or below. As the percentage of the last four answers was small, we recoded it as a doctor (1), master (2), bachelor (3), and others (4).

#### 2.3.4. Physical disease

The physical disease was evaluated by the question, “If you have been diagnosed with any physical disease?” The answer was yes (1) and no (0).

#### 2.3.5. Depression

Depression was evaluated by the Chinese version of the Center for Epidemiologic Studies-Depression Scale (CES-D) (40). In this scale, there were 20 items to evaluate the feeling of the subjects in the last week. The answer can be chosen from 0 (< 1 day), 1 (1–2 days), 2 (3–4 days), and 3 (5–7 days). The higher scores mean a higher risk of depression. The CES-D was also identified as having nice reliability and validity in the world (41, 42), and the Chinese version of the CES-D was also tested with good reliability and validity (43). In this study, Cronbach's alpha was 0.852.

### 2.4. Statistical analysis

In this study, IBM SPSS Statistics 24.0 (Web Edition) was used to conduct the data analyses. T-tests or one-way ANOVA were performed to analyze the factors associated with PV and VV. Logistic regression was conducted to further examine the factors associated with PV and VV. All the tests were two-tailed, and a *p* ≤ 0.05 was considered statistically significant.

## 3. Results

In Table 1, the descriptive analyses are shown in the second column. In the third line, 489 medical staff (23.0%) reported the experience of PV, and 1,744 (50.9%) reported the experience of VV. Single-factor analyses were also conducted to explore the factors associated with PV and VV. The factors associated with PV were gender (χ^2^ = 103.80, *p* < 0.001), age (*t* = 8.00, *p* < 0.001), marital status (χ^2^ = 7.17, *p* < 0.05), education (χ^2^ = 23.28, *p* < 0.001), physical disease (χ^2^ = 30.95, *p* < 0.001), depression (*t* = 12.52, *p* < 0.001), types of medical staff (χ^2^ = 73.46, *p* < 0.001), professional title (χ^2^ = 60.92, *p* < 0.001), manager (χ^2^ = 34.63, *p* < 0.001), working years (*t* = 6.22, *p* < 0.001), working hours/week (*t* = 8.79, *p* < 0.001), night duty frequency/week (*t* = 3.01, *p* < 0.01), monthly income (χ^2^ = 27.78, *p* < 0.001), working environment (χ^2^ = 214.19, *p* < 0.001), and social position (χ^2^ = 169.05, *p* < 0.001). Similar associated factors were also supported for VV, with additions of hospital level (χ^2^ = 9.33, *p* < 0.001) and job changing (χ^2^ = 6.24, *p* < 0.05). The detailed information can be found in Table 1.

**Table 1:** Percentage and single-factor analyses for factors associated with reported physical/verbal violence among medical staff in Shandong, China.

**Variables**	**All, *n* (%)**	**PV^†^, *n* (%)**	**VV^†^, *n* (%)**	**NV, *n* (%)**	***t/*** **χ** ^**2**^	
					**PV vs. NV**	**VV vs. NV**
Observations	3,426 (100.0)	489 (14.3)	1,744 (50.9)	1,638 (47.8)	–	–
**Gender**					103.80^***^	40.57^***^
Men	919 (26.8)	220 (23.9)	547 (59.5)	355 (38.6)		
Women	2,507 (73.2)	269 (10.7)	1,197 (47.7)	1,283 (51.2)		
Age, mean ± SD	35.14 ± 8.42	37.64 ± 8.40	36.07 ± 8.24	34.13 ± 8.53	8.00^***^	6.71^***^
**Married status**					7.17^*^	9.31^**^
Single	577 (16.8)	66 (11.4)	262 (45.4)	306 (53.0)		
Married	2,802 (81.8)	416 (14.8)	1,461 (52.1)	1,306 (46.6)		
Others	47 (1.4)	7 (14.9)	21 (44.7)	26 (55.3)		
**Education**					23.28^***^	46.10^***^
Doctor	56 (1.6)	7 (12.5)	31 (55.4)	20 (35.7)		
Master	562 (16.4)	86 (15.3)	333 (59.3)	221 (39.3)		
Bachelor	2,368 (69.1)	357 (15.1)	1,211 (51.1)	1,131 (47.8)		
Others	440 (12.8)	39 (8.9)	169 (38.4)	266 (60.5)		
**Physical disease**					30.95^***^	25.73^***^
Yes	457 (13.3)	96 (21.0)	281 (61.5)	167 (36.5)		
No	2,969 (86.7)	393 (13.2)	1,463 (49.3)	1,471 (49.5)		
Depression, mean ± SD	14.72 ± 10.38	18.50 ± 10.99	16.91 ± 10.97	12.29 ± 9.17	12.52^***^	13.23^***^
**Types of medical staff**					73.46^***^	50.87^***^
Doctor	1,268 (37.0)	253 (20.0)	740 (58.4)	504 (39.7)		
Nursing	1,695 (49.5)	179 (10.6)	776 (45.8)	904 (53.2)		
Medical technician	463 (13.5)	57 (12.3)	228 (49.2)	233 (50.3)		
**Professional title**					60.92^***^	46.50^***^
Senior	109 (3.2)	27 (24.8)	67 (61.5)	37 (33.9)		
Vice-senior	303 (8.8)	67 (22.1)	171 (56.4)	130 (42.9)		
Intermediate	1,170 (34.2)	193 (16.5)	661 (56.5)	490 (41.9)		
Junior and others	1,844 (53.8)	202 (11.0)	845 (45.8)	981 (53.2)		
**Hospital level**					0.98	9.33^**^
Level 2	1,949 (56.9)	213 (10.9)	713 (36.6)	883 (45.3)		
Level 3	1,477 (43.1)	276 (18.7)	1,031 (69.8)	755 (51.1)		
Manager					34.63^***^	19.06^***^
Yes	659 (19.2)	138 (20.9)	383 (58.1)	263 (39.9)		
No	2,767 (80.8)	351 (12.7)	1,361 (49.2)	1,375 (49.7)		
Working years, mean ± SD	47.25 ± 9.27	13.08 ± 9.12	11.73 ± 8.73	10.18 ± 9.00	6.22^***^	5.07^***^
**Job changing**					0.70	6.24^*^
Yes	438 (12.8)	62 (14.2)	247 (56.4)	185 (42.2)		
No	2,988 (87.2)	427 (14.3)	1,497 (50.1)	1,453 (48.6)		
Working hours/week, mean ± SD	47.69 ± 9.48	50.49 ± 10.85	48.87 ± 10.15	46.36 ± 8.53	8.79^***^	7.74^***^
Night duty frequency/week, mean ± SD	4.12 ± 3.55	4.45 ± 3.44	4.33 ± 3.50	3.89 ± 3.59	3.01^**^	3.59^***^
**Monthly income**					27.78^***^	40.28^***^
L1	1,615 (47.1)	191 (11.8)	729 (45.1)	862 (53.4)		
L2	1,571 (45.9)	255 (16.2)	886 (56.4)	668 (42.5)		
L3	240 (7.0)	43 (17.9)	129 (53.8)	108 (45.0)		
**Working environment**					214.19^***^	364.74^***^
Good	844 (24.6)	82 (9.7)	234 (27.7)	597 (70.7)		
Average	1,683 (49.1)	193 (11.5)	851 (50.6)	812 (48.2)		
Bad	899 (26.2)	214 (23.8)	659 (73.3)	229 (25.5)		
**Social position**					169.05^***^	241.08^***^
Good	902 (26.3)	91 (10.1)	310 (34.4)	574 (63.6)		
Average	1,934 (56.5)	249 (12.9)	985 (50.9)	925 (47.8)		
Bad	590 (17.2)	149 (25.3)	449 (76.1)	139 (23.6)		

PV, physical violence; VV, verbal violence; NV, participants without workplace violence experience; SD, standard error.

L1 denotes ≤ 5,000 RMB monthly income. L2 denotes 5,001–9,000 RMB monthly income. L3 denotes ≥9,001 RMB monthly income.

† Including the participants who experienced both physical and verbal violence.

*** p < 0.001;

** p < 0.01;

* p < 0.05.

Logistic regressions were further conducted to analyze the factors associated with PV and VV. The results supported that the factors associated with PV were male gender (OR = 1.97, *p* < 0.001), depression (OR = 1.05, *p* < 0.001), nurse (OR = 0.56, *p* < 0.01), medical technician (OR = 0.62, *p* < 0.05), manager (OR = 1.86, *p* < 0.01), night duty frequency/week (OR = 1.06, *p* < 0.01), bad working environment (OR = 2.73, *p* < 0.001), averaged social position (OR = 1.51, *p* < 0.05), and bad social position (OR = 4.21, *p* < 0.001). The factors associated with VV were male gender (OR = 1.28, *p* < 0.05), other education (OR = 0.49, *p* < 0.05), depression (OR = 1.04, *p* < 0.001), nurse (OR = 0.76, *p* < 0.05), manager (OR = 1.56, *p* < 0.001), working years (OR = 1.02, *p* < 0.05), job changing (OR = 1.33, *p* < 0.05), night duty frequency/week (OR = 1.03, *p* < 0.01), L2 income level (OR = 1.33, *p* < 0.01), averaged working environment (OR = 2.02, *p* < 0.001), bad working environment (OR = 3.52, *p* < 0.001), averaged social position (OR = 1.55, *p* < 0.001), and bad social position (OR = 3.32, *p* < 0.001). The detailed results can be found in Table 2.

**Table 2:** Logistic regression analyses for the factors associated with physical violence among medical staff in Shandong, China [OR (95% CI)].

**Variables**	**PV^†^vs. NV**	**VV^†^vs. NV**
Observations	489 vs. 1638	1744 *vs*. 1638
Men	1.97 (1.47, 2.64)^***^	1.28 (1.05, 1.57)^*^
Age	1.02 (0.99, 1.05)	1.00 (0.98, 1.02)
**Married status (ref**. = **single)**		
Married	0.78 (0.54, 1.13)	0.86 (0.69, 1.08)
Others	0.60 (0.21, 1.74)	0.67 (0.34, 1.32)
**Education (ref**. = **doctor)**		
Master	0.71 (0.26, 1.96)	0.68 (0.36, 1.30)
Bachelor	0.93 (0.34, 2.54)	0.63 (0.34, 1.20)
Others	0.61 (0.21, 1.80)	0.49 (0.25, 0.96)^*^
Physical disease	0.99 (0.70, 1.39)	1.00 (0.79, 1.27)
Depression	1.05 (1.04, 1.07)^***^	1.04 (1.03, 1.04)^***^
**Types of medical staff (ref**. = **doctor)**		
Nursing	0.56 (0.39, 0.80)^**^	0.76 (0.60, 0.96)^*^
Medical technician	0.62 (0.41, 0.92)^*^	0.89 (0.69, 1.15)
**Professional title (ref**. = **senior)**		
Vice-senior	0.86 (0.43, 1.72)	0.85 (0.51, 1.43)
Intermediate	1.06 (0.51, 2.21)	1.20 (0.70, 2.04)
Junior and others	0.98 (0.42, 2.29)	1.16 (0.64, 2.10)
Level 2 hospital (ref. = level 3)	0.91 (0.70, 1.18)	0.91 (0.77, 1.07)
Manager	1.86 (1.29, 2.67)^**^	1.56 (1.22, 1.99)^***^
Working years	1.02 (1.00, 1.04)	1.02 (1.00, 1.04)^*^
Job changing	1.11 (0.76, 1.63)	1.33 (1.05, 1.70)^*^
Working hours/week	1.01 (1.00, 1.02)	1.01 (1.00, 1.02)
Night duty frequency/week	1.06 (1.02, 1.10)^**^	1.03 (1.01, 1.06)^**^
**Monthly income (ref**. = **L1)**		
L2	1.26 (0.96, 1.66)	1.33 (1.11, 1.59)^**^
L3	1.02 (0.59, 1.78)	1.12 (0.77, 1.62)
**Working environment (ref**. = **good)**		
Average	1.20 (0.87, 1.66)	2.02 (1.66, 2.46)^***^
Bad	2.73 (1.88, 3.96)^***^	3.52 (2.75, 4.51)^***^
**Social position (ref**. = **good)**		
Average	1.51 (1.10, 2.07)^*^	1.55 (1.28, 1.88)^***^
Bad	4.21 (2.78, 6.39)^***^	3.32 (2.51, 4.40)^***^
Constant	0.02^***^	0.13^***^
R^2^	0.32	0.24

PV, physical violence; VV, verbal violence; NV, participants without workplace violence experience; OR, odd ratio; CI, confidential interval.

L1 denotes ≤ 5,000 RMB monthly income. L2 denotes 5,001–9,000 RMB monthly income. L3 denotes ≥9,001 RMB monthly income.

† Including the participants who experienced both physical violence and verbal violence.

*** p < 0.001;

** p < 0.01;

* p < 0.05.

## 4. Discussion

In this study, there were several critical findings. First, we found that more than half of the medical staff (52.2%) experienced WPV. The percentage for PV against medical staff was 14.3%, and it was 50.9% for VV. Second, both PV and VV were associated with several work-related variables, such as types of medical staff, manager, night duty frequency, self-reported working environment, and self-reported social position. The other risk factors were male gender and depression. Third, VV was positively associated with working years, job changing, and monthly income. However, these work-related variables were not supported as being associated with PV.

The first finding in this study was about the prevalence of WPV, PV, and VV among medical staff, and we found that 52.2% of medical staff reported WPV, 14.3% of medical staff reported PV, and 50.9% of medical staff reported VV. Compared with other studies, this prevalence of WPV, PV, and VV was slightly lower than that in other studies. This prevalence of WPV was 54.8% among nurses in Turkey (44) and 71.9% among medical staff at primary hospitals (45). For the prevalence of PV and VV, they were also ~20% and 70%, respectively (46, 47). One of the explanations was about the different current situations of WPV among doctors, nurses, and medical technicians (48). In this study, we interviewed medical technicians who have a lower prevalence of WPV (49). The other reason may be explained by cultural differences in the perception of WPV in different countries. Harmonization is one of the characteristics of Chinese Confucian culture (50). Shandong Province was also the headstream of Confucian culture, which was also deeply influenced by this culture. This kind of harmonization may also reduce the occurrence of WPV against medical staff.

In this study, a higher prevalence of PV and VV was reported among doctors and managers, which was also supported in previous studies (32, 47, 51). Doctors need to take charge of the therapeutic plan and frequently communicate with patients. Dissatisfaction with the professional diagnosis and treatment processes was one of the main reasons for hospital violence (52), and it may explain why doctors are at a higher risk of WPV than nurses and medical technicians. Managers need to deal with the problems of patients in their departments, and they frequently communicate with patients and their relatives about healthcare problems. This means that they are also at higher risk of PV and VV.

The other finding in this study was about the associations between night duty frequency, self-reported working environment, self-reported social position, and WPV (including PV and VV). The positive association between more night duty frequency and WPV was also supported by previous studies (29). One of the explanations was about the identified associated factors, such as job burnout (53) and poor sleep quality (54). Some studies supported the fact that most WPV happened at night (55). The findings in the study also supported the fact that a bad self-reported working environment and a bad self-reported social position were also positively associated with WPV. They may have a bidirectional causal relationship. Medical staff who experienced WPV may feel a worse working environment or social position. Medical staff with the feeling of a worse working environment and social position may be at a higher risk of psychological health, which is also a risk factor for WPV (56, 57).

We also found that VV was positively associated with working years and monthly income, but both were not supported as being associated with PV. In this study, VV was evaluated in a lifetime. Medical staff with longer work years were also in longer communication with the patients and their relatives, and they were also at higher risk of VV. The positive association between monthly income level and VV may be explained by the more diagnosis and treatment chance. In China, the income of the medical staff is positively associated with the number of health services, and more quantity of health services increases the risk of VV among the patients and their relatives. However, when we went to PV, the associations were not supported in this study. One of the reasons may be the serious consequences of PV (9, 58, 59). The patients and their relatives may also be cautious about conducting PV. However, previous studies supported the fact that PV is mainly caused by dissatisfaction with the attitude of the medical staff (34). Medical staff with long working years may have experience with the attitude, and it may weaken the association between working years and PV.

We also found that job changing was positively associated with VV but not with PV. Some studies supported the associations between job changing and workplace violence (60). This association may be explained by job satisfaction. Previous studies identified that medical staff with low job satisfaction were at higher risk of workplace violence (61, 62), and it also increased their intention to leave (63). However, this study did not support the association between job changing and PV. One of the reasons may be the small sample size for PV. The other reason may be mental stress from the PV experiences. Medical staff with PV experiences may feel mental stress, and some working environment change may be helpful for them to reduce the mental stress, and job changing may be one method of changing the working environment.

Both gender and depression were also associated with WPV in this study. For gender, we found that the female gender was at a lower risk of experiencing WPV compared with the male gender. This is different from other findings in Western countries, which found female health workers were at higher risk of WPV (64). This may be explained by the Confucian culture of the weak female gender in China (65). Medical staff with depression may find it hard to supply good quality medical services, and it may further result in WPV. In contrast, depression may also be one of the negative outcomes of WPV, which was supported by previous studies (58).

Although there were some significant findings in this study, several limitations should be considered when we interpret the results. First, we cannot get any causal relationships for the association between work-related variables and WPV because of the cross-sectional design. Second, all the factors analyzed in this study were collected by self-reporting of medical staff, and it may also bring some bias to the findings in this study. Third, the data analyzed in this study were collected from several general hospitals in Shandong Province, China. The multiple stratified random cluster sampling method may overrepresent the situations in economically developed cities, and we should also be cautious when extending the findings to other regions or countries.

## 5. Conclusion

In this study, we found that both PV and VV had a higher prevalence among medical staff in Shandong, China. Both PV and VV were positively associated with doctor, manager, more night duty frequency, perceived bad working environment, or social position. VV was positively associated with more working years, job changing, and more monthly income, but they were not supported to be associated with PV. These findings remind us that work-related variables were associated with WPV, and there were some different associated factors between PV and VV. Some special strategies about the work-related variables should be applied for controlling WPV, and the differences between PV and VV should also arouse our attention.

## Data availability statement

The raw data supporting the conclusions of this article will be made available by the authors, without undue reservation.

## Ethics statement

The studies involving human participants were reviewed and approved by the Institutional Review Board of Shandong University School of Public Health. The patients/participants provided their written informed consent to participate in this study.

## Author contributions

LS analyzed the data and drafted the manuscript. WZ collected the data and comment on the draft of this manuscript. AC designed the study and revised the draft. All authors read and approved the final manuscript.
